# Amelioration of Hyperglycemia with a Sodium-Glucose Cotransporter 2 Inhibitor Prevents Macrophage-Driven Atherosclerosis through Macrophage Foam Cell Formation Suppression in Type 1 and Type 2 Diabetic Mice

**DOI:** 10.1371/journal.pone.0143396

**Published:** 2015-11-25

**Authors:** Michishige Terasaki, Munenori Hiromura, Yusaku Mori, Kyoko Kohashi, Masaharu Nagashima, Hideki Kushima, Takuya Watanabe, Tsutomu Hirano

**Affiliations:** 1 Department of Medicine, Division of Diabetes, Metabolism, and Endocrinology, Showa University School of Medicine, Hatanodai, Shinagawa-ku, Tokyo, Japan; 2 Laboratory of Cardiovascular Medicine, Tokyo University of Pharmacy and Life Sciences, Horinouchi, Hachioji-City, Tokyo, Japan; University of Catanzaro Magna Graecia, ITALY

## Abstract

Direct associations between hyperglycemia and atherosclerosis remain unclear. We investigated the association between the amelioration of glycemia by sodium-glucose cotransporter 2 inhibitors (SGLT2is) and macrophage-driven atherosclerosis in diabetic mice. We administered dapagliflozin or ipragliflozin (1.0 mg/kg/day) for 4-weeks to apolipoprotein E-null (*Apoe* ^*−/−*^) mice, streptozotocin-induced diabetic *Apoe* ^*−/−*^ mice, and diabetic *db/db* mice. We then determined aortic atherosclerosis, oxidized low-density lipoprotein (LDL)-induced foam cell formation, and related gene expression in exudate peritoneal macrophages. Dapagliflozin substantially decreased glycated hemoglobin (HbA1c) and glucose tolerance without affecting body weight, blood pressure, plasma insulin, and lipids in diabetic *Apoe* ^*−/−*^ mice. Aortic atherosclerotic lesions, atheromatous plaque size, and macrophage infiltration in the aortic root increased in diabetic *Apoe* ^*−/−*^ mice; dapagliflozin attenuated these changes by 33%, 27%, and 20%, respectively. Atherosclerotic lesions or foam cell formation highly correlated with HbA1c. Dapagliflozin did not affect atherosclerosis or plasma parameters in non-diabetic *Apoe* ^*−/−*^ mice. In *db/db* mice, foam cell formation increased by 4-fold compared with C57/BL6 mice, whereas ipragliflozin decreased it by 31%. Foam cell formation exhibited a strong correlation with HbA1c. Gene expression of lectin-like ox-LDL receptor-1 and acyl-coenzyme A:cholesterol acyltransferase 1 was upregulated, whereas that of ATP-binding cassette transporter A1 was downregulated in the peritoneal macrophages of both types of diabetic mice. SGLT2i normalized these gene expressions. Our study is the first to demonstrate that SGLT2i exerts anti-atherogenic effects by pure glucose lowering independent of insulin action in diabetic mice through suppressing macrophage foam cell formation, suggesting that foam cell formation is highly sensitive to glycemia *ex vivo*.

## Introduction

Diabetes accelerates the clinical course of atherosclerosis, a condition associated with arterial endothelial dysfunction and several metabolic abnormalities, can create a pro-inflammatory environment, and can induce foam cell formation of macrophages [1–3]. Because atherosclerosis is accelerated in both type 1 and type 2 diabetes [4–6], it is reasonable to assume that hyperglycemia plays a major role in the pathogenesis of diabetes-induced atherosclerosis. While, patients with metabolic syndrome or pre-diabetes without significant hyperglycemia often develop atherosclerotic cardiovascular events [7–9]. However, it remains unclear whether hyperglycemia contributes to the development of atherosclerosis in diabetes because many risk factors change with the impairment of insulin action. There have been numerous *in vitro* studies that have examined the toxic effects of high glucose levels on vascular and inflammatory cells related to atherosclerotic lesions [10–12]. However, studies on cultured cells have provided only hypothesis-generating results that need to be evaluated in *in vivo* studies.

Recently, sodium-glucose cotransporter 2 inhibitors (SGLT2is) have been developed as novel therapeutic agents for the treatment of patients with type 2 diabetes. These drugs inhibit the reabsorption of glucose in the proximal tubules of the kidney, leading to increased urinary glucose excretion and amelioration of hyperglycemia in patients with diabetes [13–16]. This simple mechanism for lowering glucose levels does not directly influence any insulin-related metabolic changes. This agent could be a useful tool to study the direct relationship between glycemia and atherosclerosis independent of insulin secretion and action *in vivo*. The acceleration of atherosclerosis is influenced by abnormalities in cellular cholesterol homeostasis, which is demonstrated by the subendothelial accumulation of lipid-laden macrophage foam cells. The accumulated foam cells in the subendothelial space create lipid-rich plaque, which is an initial process of atherosclerosis [1–3]. There have been several *in vitro* studies that have revealed that high glucose levels enhance foam cell formation in cultured macrophages [17, 18]; however, it remains unclear whether foam cell formation measured *ex vivo* reflects atherosclerotic lesions. Foam cell formation is regulated by several factors: 1) scavenger receptors, such as CD36 and lectin-like ox- low-density lipoprotein (LDL) receptor-1 (Lox-1) [1], 2) acyl-coenzyme A:cholesterol acyltransferase 1 (ACAT1), a rate-limiting enzyme for the esterification of cholesterol [1], and 3) free-cholesterol efflux mediated by ATP-binding cassette transporter A1 (ABCA1) and ATP-binding cassette sub-family G member 1 (ABCG1) [1]. Several *in vitro* studies have revealed that scavenger receptors were upregulated and that either ABCA1 or ABCG1 was downregulated in high glucose conditions [19, 20]. The aim of the present study was to evaluate the effect of the amelioration of hyperglycemia by SGLT2i on the development of aortic atherosclerotic lesions, macrophage foam cell formation, and related molecules in type 1 and type 2 diabetic mice.

## Materials and Methods

### Chemicals and reagents

Streptozotocin (STZ) was purchased from Sigma-Aldrich (Saint-Louis, MO, USA). Dapagliflozin and ipragliflozin were kindly gifted from AstraZeneca (Gaithersburg, MD, USA) and Astellas Pharma, Inc. (Tokyo, Japan), respectively.

### Animal experiments

This study was conducted in strict accordance with the recommendations in the Guide for the Care and Use of Laboratory Animals of the National Institutes of Health. The protocol was approved by the Institutional Animal Care and Use Committee of Showa University (Permit Number: 04090). All surgeries and sacrifice were performed under general anesthesia using isoflurane, and all efforts were made to minimize suffering. The health condition of all mice was carefully checked every day by us or animal experts in the animal facility of Showa University School of Medicine.

### Experiment #1

A total of forty-five male apolipoprotein E-null (*Apoe* ^*−/−*^) mice were purchased from Sankyo Labo Service (Tokyo, Japan) at the age of 9 weeks and were kept on a standard rodent chow until the age of 15 weeks. From 15 weeks of age, the animals received intraperitoneal injections of saline or streptozotocin (50 mg/kg/day) for 5 consecutive days to create a type 1 diabetes model, according to the method described by Takeda *et al* [21]. Adverse effects of STZ injection in mice has been reported as follows: weight loss, respiratory distress, rapid glycemic shifts resulting in life-threatening hypoglycemia, and a generalized poor body condition [22]. In our protocol, a mouse that lost weight by more than 20% from the baseline, or that showed obvious weakness undergoes euthanasia with CO_2_ exposure to avoid severe pain or distress. Three mice found dead in their cages within 7 days since the last injection of STZ. The other mice did not show any clinical signs including severe weight loss or obvious weakness. The fasting blood glucose (FBG) levels were measured 10 days after the last injection *via* a tiny cut in the tail vein using the glucose monitoring system (Nipro StatStrip; Nipro Corporation, Osaka, Japan). All survived mice showed fasting blood glucose levels higher than 200 mg/dL, and were enrolled in this experiment as diabetic mice. At the age of 17 weeks, both non-diabetic and diabetic mice were switched to an atherogenic diet containing 30% fat, 20% sucrose, 8% NaCl, and 0.15% cholesterol (Oriental Yeast, Tokyo, Japan) [23–26] and randomly assigned to vehicle or SGLT2i treatment. The SGLT2i (dapagliflozin) was given *via* drinking water at a dose of 1.0 mg/kg/day. After 4 weeks of this treatment, peritoneal macrophages were harvested, and the mice were sacrificed to collect the aorta under general anesthesia with isoflurene.

### Experiment #2

Thirty-one male *db/db* mice (a mouse model of type 2 diabetes) and seven male C57/BL6 (wild type) mice were purchased from Sankyo Labo Service at the age of 6 weeks and kept on standard rodent chow. All *db/db* mice developed diabetes at the age of 8 weeks with FBG levels above 200 mg/dL. Starting from the age of 9 weeks, the diabetic *db/db* mice were given standard rodent chow with or without diet containing the SGLT2i (ipragliflozin) at the dose of 1.0 mg/kg/day for 4 weeks. The wild-type mice were kept on standard rodent chow; they were used as the control model during the same period. At the end of the treatment period, we collected peritoneal macrophages.

### Measurements

Glycated hemoglobin (HbA1c) levels were measured through a cut in the tail vein using the quick test A1C Now Plus (Bayer, Frankfurt, Germany) before sacrificing the animals. The blood samples were collected after a 6-h fast. The plasma levels of glucose, total cholesterol, high-density lipoprotein (HDL) cholesterol, and triglyceride were measured by enzymatic methods (WAKO, Osaka, Japan). We had previously reported that dipeptidyl peptidase-4 inhibitor elevated active glucagon-like peptide-1 (GLP-1) and total glucose-dependent insulinotropic polypeptide (GIP), that prevented the acceleration of atherosclerosis by suppressing foam cell formation in *Apoe* ^*−/−*^ mice [22, 23]. Therefore, we measured the endogenous incretin levels in this study. The plasma levels of insulin, active GLP-1, and total GIP were determined by enzyme-linked immunosorbent assay (ELISA) (Ultra-sensitive mouse insulin ELISA kit from Morinaga, Kanagawa, Japan; GLP-1 (active) ELISA and Rat/Mouse GIP (total) ELISA from EMD Millipore (Billerica, MA, USA).

### Oral glucose tolerance test (OGTT)

Glucose (0.5 g/kg body weight) was orally administered through a gavage tube after 6 h of fasting, and the blood glucose levels were measured at the specified time points of 0 (pre-glucose/fasting glucose level) and at 15, 30, 60, and 120 min after using the Nipro StatStrip. The area under the curve (AUC) was calculated as the area under the glucose curve from 0 to 120 min multiplied by the minutes at the measured time points.

### Blood pressure measurement

Blood pressure and pulse were measured on the day of sacrifice in the fasting state using the tail-cuff method (Model MK-2000ST, Muromachi-Kikai, Tokyo, Japan).

### Atherosclerotic lesion assessment

The whole aorta was washed with perfused phosphate buffered saline (PBS) and fixed with 4% paraformaldehyde (wt/vol). After surrounding connective and adipose tissues were carefully removed, the aorta was longitudinally dissected and excised from the root to the bifurcation of the iliac artery. The entire aorta and cross-sections of the aortic root were stained with oil red O for the assessment of atherosclerotic lesions [23–27]. Macrophage infiltration into the aortic wall was visualized using anti-mouse MOMA-2 antibody staining [23–27]. The en face oil red O positive area was traced by an investigator that was blind to the treatment and measured using an image analyzer (Adobe Photoshop, San Jose, CA, USA; Image J Software, Bethesda, MD, USA). The severity of atheromatous plaques and lesion area of macrophage infiltration were assessed as the sum of stained areas in the aortic root [23–27].

### Cell culture

Four days after the intraperitoneal injection of 4 mL of aged autoclaved thioglycolate broth, exudate peritoneal cells were isolated from treated non-diabetic or diabetic *Apoe* ^*−/−*^ mice at 21 weeks of age or from diabetic *db/db* mice at 13 weeks of age using a peritoneal lavage with 8 mL of ice-cold PBS [23–26]. The cells were suspended in a culture medium (RPMI-1640 supplemented with 10% FBS, streptomycin, and penicillin) and seeded onto 3.5-cm dishes (3×10^6^ cells/dish) for either a reverse transcription polymerase chain reaction (RT-PCR) or a cholesterol esterification assay. After 1 h of incubation at 37°C in a 5% CO_2_ incubator to allow cell adhesion to the dish, the medium was discarded to remove any non-adherent cells, and the adherent cells were identified as peritoneal macrophages [23–26].

### Cholesterol esterification assay

The adherent macrophages were incubated for 18 h in the RPMI-1640 medium that contained 10 μg/mL human oxidized (ox)-LDL with 0.1 mmol/L [^3^H]oleate conjugated with bovine serum albumin. The cellular lipids were extracted and the radioactivity of the cholesterol [^3^H]oleate was determined with thin-layer chromatography [23–26].

### Gene expression

Total RNA was extracted from peritoneal macrophages using a commercially available kit (QIAGEN; Hilden, Germany). The gene expressions were assessed by real-time RT-PCR using the TaqMan gene expression assay and a sequence detection system (ABI PRISM 7900, Life Technologies, Carlsbad, CA, USA), which used cDNA synthesized from isolated RNA samples to detect the expression of target genes, such as Lox-1; Mm00454586_m1, CD36; Mm01135198_ml, ACAT1; Mm00507463_ml, ABCA1; Mm00442646_ml, and ABCG1; Mm00437390_m1. TaqMan gene expression assays for mouse glyceraldehyde-3-phosphate dehydrogenase (GAPDH) was purchased from Life Technologies. The levels of these gene expressions were normalized using GAPDH as an internal control.

### Statistical analysis

All values are expressed as mean ± standard error of mean (SEM). We used one-way analysis of variance (ANOVA) followed by Tukey test for the comparison of more than two groups and the unpaired t-test for the comparison of two groups. The correlation between two valuables was determined using Pearson’s correlation test. Statistical calculations were performed using JMP software (Version 11; SAS Institute Inc., Cary, NC, USA). The significance level was defined as *p* < 0.05.

## Results

### Characteristics and laboratory data

Table 1 shows the characteristics and laboratory data from four groups of non-diabetic and diabetic *Apoe* ^−/−^ mice that did or did not receive SGLT2i (dapagliflozin) (Experiment #1). Although the systolic blood pressure (SBP) was slightly decreased in dapagliflozin-treated diabetic *Apoe* ^−/−^ mice, this change was not statistically significant. Non-diabetic *Apoe* ^−/−^ mice that received dapagliflozin, diabetic mice that received vehicle, and diabetic mice that received dapagliflozin consumed 1.4–1.7 times more drinking water and produced 1.7–2.6 times more urine than non-diabetic mice that received vehicle. These diabetic *Apoe* ^−/−^ mice exhibited the classical features of STZ-induced insulin-deficient type 1 diabetes, such as severe hyperglycemia, hyperphagia, low body weight gain, and low insulin concentration. Based on our preliminary experiments with the same protocol used in the present study, we expected a successful induction rate of diabetes in more than 90% of mice, and a mortality rate including euthanasia in less than 10%. In the present study, all the mice except for three dead ones developed diabetes, showing the successful rate of 93% and the mortality rate of 7%. There is one previous study, which used the same administration protocol as ours, reporting the mortality rate of approximately 8% [28], which are comparable with our results. The diabetic mice that received vehicle had slightly elevated levels of total cholesterol compared with their non-diabetic counterparts but this was not statistically significant. Diabetic *Apoe* ^−/−^ mice lost 10% (*p* < 0.05) of their body weight compared with non-diabetic *Apoe* ^−/−^ mice. In diabetic *Apoe* ^−/−^ mice, dapagliflozin had no effect on insulin levels after 6 h of fasting, but it significantly decreased FBG levels from 417 ± 31 mg/dL to 134 ± 18 mg/dL and HbA1c levels from 8.2% ± 0.3% to 5.8% ± 0.3%. However, there were no changes in the plasma levels of active GLP1, total GIP, total cholesterol, triglyceride, and HDL cholesterol among the groups.

**Table 1:** Characteristics and laboratory data from four groups of non-diabetic *Apoe* ^−/−^ mice and STZ-induced diabetic *Apoe* ^−/−^ mice treated with or without dapagliflozin.

	*Apoe* ^−/−^ mice		Diabetic *Apoe* ^*−/−*^ mice	
	Vehicle	Dapagliflozin	Vehicle	Dapagliflozin
Number	14	6	15	7
Final body weight (g)	28.3 ± 3.0	28.4 ± 2.4	25.4 ± 1.7 a,b	25.6 ± 1.7 a,b
Food intake (g/day)	6.9 ± 0.6	8.1 ± 0.7 a	8.3 ± 0.9 a	8.2 ± 0.7 a
Water intake (mL/day)	10.4 ± 0.8	14.2 ± 1.0 a	15.8 ± 2.1 a	17.2 ± 1.5 a
Urine volume (mL/day)	6.9 ± 0.9	11.9 ± 1.2 a	14.1 ± 1.8 a	18.1 ± 1.7 a
SBP (mmHg)	101 ± 4	100 ± 6	100 ± 5	91 ± 6
Pulse (/min)	515 ± 28	498 ± 24	535 ± 19	511 ± 21
Glucose (mg/dL)	117 ± 18	136 ± 16	417 ± 31 a,b	134 ± 18 c
HbA1c (%)	4.6 ± 0.1	4.6 ± 0.1	8.2 ± 0.3 a,b	5.8 ± 0.3 a,b,c
Insulin (ng/mL)	0.99 ± 0.06	0.78 ± 0.16	0.24 ± 0.06 a,b	0.26 ± 0.05 a,b
Total-C (mg/dL)	523 ± 71	486 ± 56	662 ± 66	580 ± 27
HDL-C (mg/dL)	13 ± 2	14 ± 2	10 ± 1	9 ± 2
Triglyceride (mg/dL)	58 ± 9	49 ± 12	71 ± 15	45 ± 8
Active GLP-1 (pmol/L)	3.9 ± 0.6	5.0 ± 1.2	6.0 ± 0.7	4.1 ± 0.8
Total GIP (pmol/L)	41 ± 5	62 ± 29	30 ± 4	42 ± 5

Non-diabetic mice that received vehicle, non-diabetic mice that received dapagliflozin, diabetic mice that received vehicle, diabetic mice that received dapagliflozin were measured. The values show mean ± SEM. dapagliflozin, sodium glucose co-transporter 2 inhibitor; SBP, systolic blood pressure; HbA1c, hemoglobin A1c; Total-C, total cholesterol; HDL-C, high-density lipoprotein cholesterol; GLP-1, glucagon like peptide-1; GIP, glucose-dependent insulinotropic polypeptide. One-way ANOVA followed by Tukey test:

^a,^ *p* < 0.05 vs. non-diabetic mice that received vehicle;

^b,^ *p* < 0.05 vs. non-diabetic mice that received dapagliflozin;

^c,^ *p* < 0.05 vs. diabetic mice that received vehicle.

Table 2 shows the metabolic parameters of diabetic *db/db* mice with or without SGLT2i (ipragliflozin) compared with wild-type mice (Experiment #2). As a result of severe hyperglycemia, diabetic *db/db* mice consumed approximately 1.3 times more drinking water, had approximately 1.4 times more food intake, and passed 2 times the urine volume compared with wild-type mice. These diabetic *db/db* mice exhibited the classical features of type 2 diabetes, such as severe hyperglycemia, high insulin concentrations, and high lipid profiles (total cholesterol, HDL cholesterol, and triglyceride). In diabetic *db/db* mice, SBP was lower in the ipragliflozin-treated group, but this change was not statistically significant. After ipragliflozin treatment, mice consumed approximately 2.4 times more drinking water and produced approximately 1.6 times more urine than those that received vehicle. These diabetic *db/db* mice that received ipragliflozin lost 6% (*p* < 0.05) of their body weight compared with those that received vehicle. Ipragliflozin significantly decreased HbA1c levels from 8.0% ± 0.2% to 6.4% ± 0.3% and FBG levels from 326 ± 32 mg/dL to 180 ± 29 mg/dL. There were no changes in the plasma levels of active GLP1, total GIP, total cholesterol, HDL cholesterol, and triglyceride with or without ipragliflozin treatment in diabetic *db/db* mice.

**Table 2:** Characteristics and laboratory data from three groups of diabetic *db/db* mice with or without ipragliflozin, and wild-type mice that received vehicle.

	C57/BL6 mice	Diabetic *db/db* mice	
	Vehicle	Vehicle	Ipragliflozin
Number	7	18	13
Final body weight (g)	25.2±0.3	49.6 ± 0.6 a	46.8 ± 0.7 a,b
Food intake (g/day)	3.1±0.2	4.4 ± 0.3 a	4.8 ± 0.4 a
Water intake (mL/day)	3.2±0.3	4.0 ± 0.4	9.4 ± 1.1 a,b
Urine volume (mL/day)	3.8±0.4	7.6±0.3 a	12.5 ±1.2 a,b
SBP (mmHg)	102±5	109 ± 2	104 ± 3
Pulse (/min)	546±21	609± 19	633± 13
Glucose (mg/dL)	101±11	326 ± 32 a	180±29 a,b
HbA1c (%)	4.2±0.1	8.0 ± 0.2 a	6.4 ± 0.3 a,b
Insulin (ng/mL)	0.63±0.17	6.36±0.62 a	5.75± 0.9 a
Total-C (mg/dL)	78±8	134 ± 8 a	171± 19 a
HDL-C (mg/dL)	30±4	63± 8 a	55±6 a
Triglyceride (mg/dL)	20±3	67±7 a	60±8 a
Active GLP-1 (pmol/L)	1.67±0.2	3.13±0.05 a	3.7 ± 0.9 a
Total GIP (pmol/L)	24.5±3.9	51.2±5.5 a	45.4±9.9 a

Non-diabetic wild-type mice that received vehicle, diabetic *db/db* mice that received vehicle, diabetic *db/db* mice that received ipragliflozin were measured. The values show mean ± SEM. Ipragliflozin, sodium glucose co-transporter 2 inhibitor.

One-way ANOVA followed by Tukey test:

^a,^ *p* < 0.05 vs. wild-type mice that received vehicle;

^b,^ *p* < 0.05 vs. diabetic *db/db* mice that received ipragliflozin.

### Glucose tolerance

**Fig 1:**
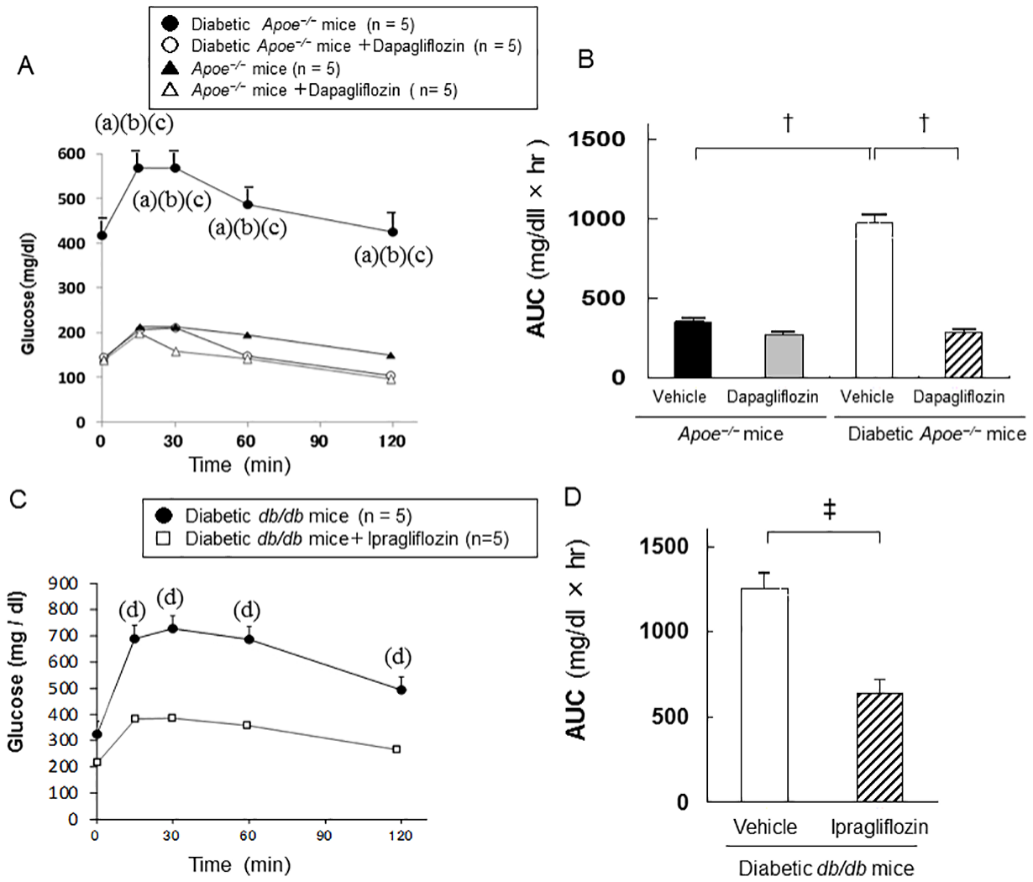
Oral glucose tolerance test. Glucose (0.5 g/kg body weight) was administered orally through a gavage tube after 6 h of fasting, and blood glucose levels were measured at the specified time points of 0 (pre-glucose/fasting glucose level), and at 15, 30, 60, and 120 min after administration. Glucose curve after oral glucose loading in non-diabetic and diabetic apolipoprotein E-null mice (*Apoe* ^*−/−*^) mice that received vehicle or dapagliflozin (A), and the area under the curve (AUC) (B). Glucose curve after oral glucose loading in diabetic *db/db* mice that received vehicle or ipragliflozin (C), and the AUC (D). The data are expressed as mean ± SEM. One-way analysis of variance (ANOVA) followed by Tukey test for the comparison of *Apoe* ^*−/−*^ mice: a, *p* < 0.05 vs. non-diabetic *Apoe* ^*−/−*^ mice that received vehicle; b, *p* < 0.05 vs. non-diabetic *Apoe* ^*−/−*^ mice that received dapagliflozin; c, *p* < 0.05 vs. diabetic *Apoe* ^*−/−*^ mice that received dapagliflozin. Unpaired t-test for the comparison of *db/db* mice: d, *p* < 0.05 vs. vehicle. n = 5 per group. ^†^ *p* < 0.01, ^‡^ *p* < 0.001.

In a subset of animals, OGTT was conducted to determine glucose tolerance (Fig 1). Diabetic *Apoe* ^*−/−*^ mice had a significantly higher glucose loading after OGTT and AUC (975 ± 46 mg/dL×h) than non-diabetic *Apoe* ^*−/−*^ mice (AUC; 356 ± 20 mg/dL×h). The treatment with dapagliflozin improved glucose loading after OGTT and improved AUC (291 ± 16 mg/dL×h) in diabetic *Apoe* ^*−/−*^ mice compared with those that received vehicle. In diabetic *db/db* mice, mice treated with ipragliflozin had significantly lower glucose loading after OGTT and a lower AUC (637 ± 28 mg/dL×h) compared with those that received vehicle (AUC; 1255 ± 108 mg/dL×h) as well.

### Atherosclerotic lesions assessment

**Fig 2:**
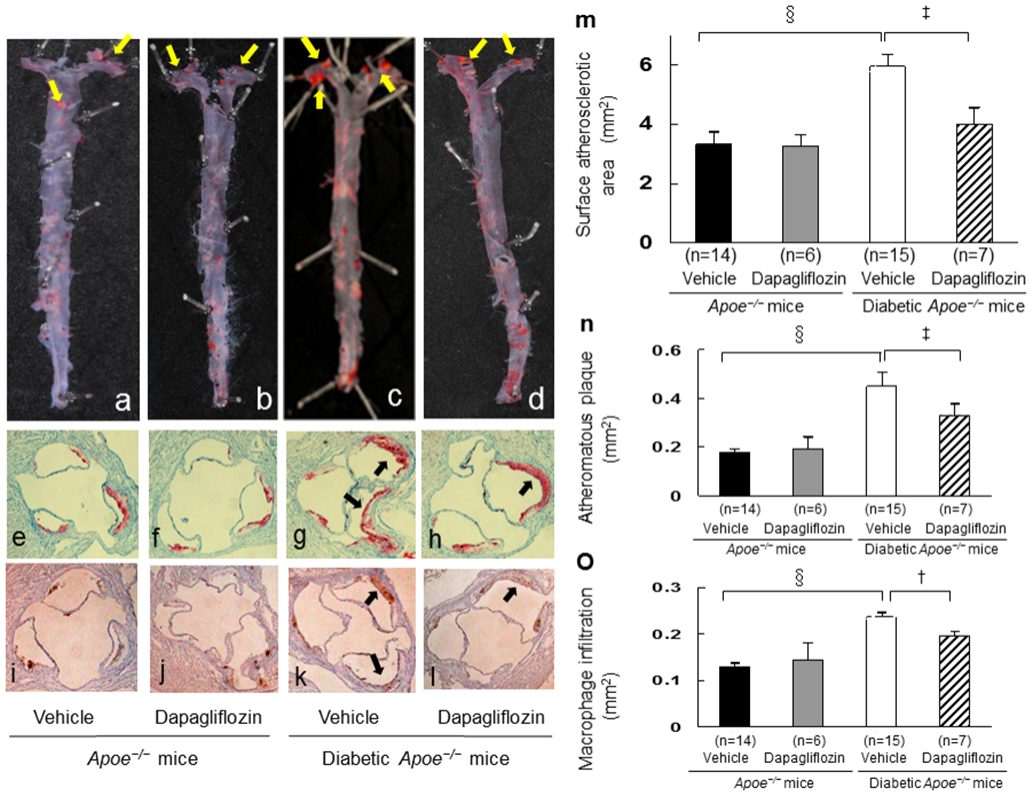
Suppressive effects of dapagliflozine administration against the development of aortic atherosclerotic lesions in non-diabetic and diabetic *Apoe* ^*−/−*^ mice. Twenty-two mice at 15 weeks of age were made diabetic with peritoneal injections of STZ (50 mg/kg/day) for 5 consecutive days and twenty mice were treated with saline. The 17-week-old non-diabetic and diabetic *Apoe* ^*−/−*^ mice were orally given SGLT2i (dapagliflozin) or vehicle for 4 weeks, starting from 17 weeks of age. Representative atherosclerotic lesions in the aortic surface stained with oil red O (a-d) and measured (m). Yellow arrows show notable atherosclerotic lesions. In the aortic root, the atheromatous plaques and monocyte/macrophage accumulations were stained with Oil red O (e-h) or anti-MOMA2 antibody (i-l). Black arrows show notable atheromatous plaques. The severity of atheromatous plaques (n) and degree of monocyte/macrophage accumulation (o) were evaluated. The data are expressed as mean ± SEM. One way ANOVA followed by Tukey test: ^†^ *p* < 0.01, ^‡^ *p* < 0.001, ^§^ *p* < 0.0001.

Atherosclerotic lesions were significantly accelerated in diabetic *Apoe* ^*−/−*^ mice. The surface areas of atherosclerotic lesions, atheromatous plaque size, and macrophage infiltration in the aortic root were increased by 78%, 150%, and 82% compared with those in non-diabetic *Apoe* ^*−/−*^ mice, suggesting the importance of hyperglycemia as an enhancer of atherosclerotic lesion formation. Diabetic *Apoe* ^*−/−*^ mice treated with dapagliflozin showed significant reductions in the surface areas of atherosclerotic lesions (33%, *p* < 0.001), in atheromatous plaque size (27%, *p* < 0.001), and in macrophage infiltration (20%, *p* < 0.01) compared with those that received vehicle. In contrast, dapagliflozin did not show any effect on atherosclerosis in non-diabetic *Apoe* ^*−/−*^ mice (Fig 2).

### Foam cell formation in exudate peritoneal macrophages

**Fig 3:**
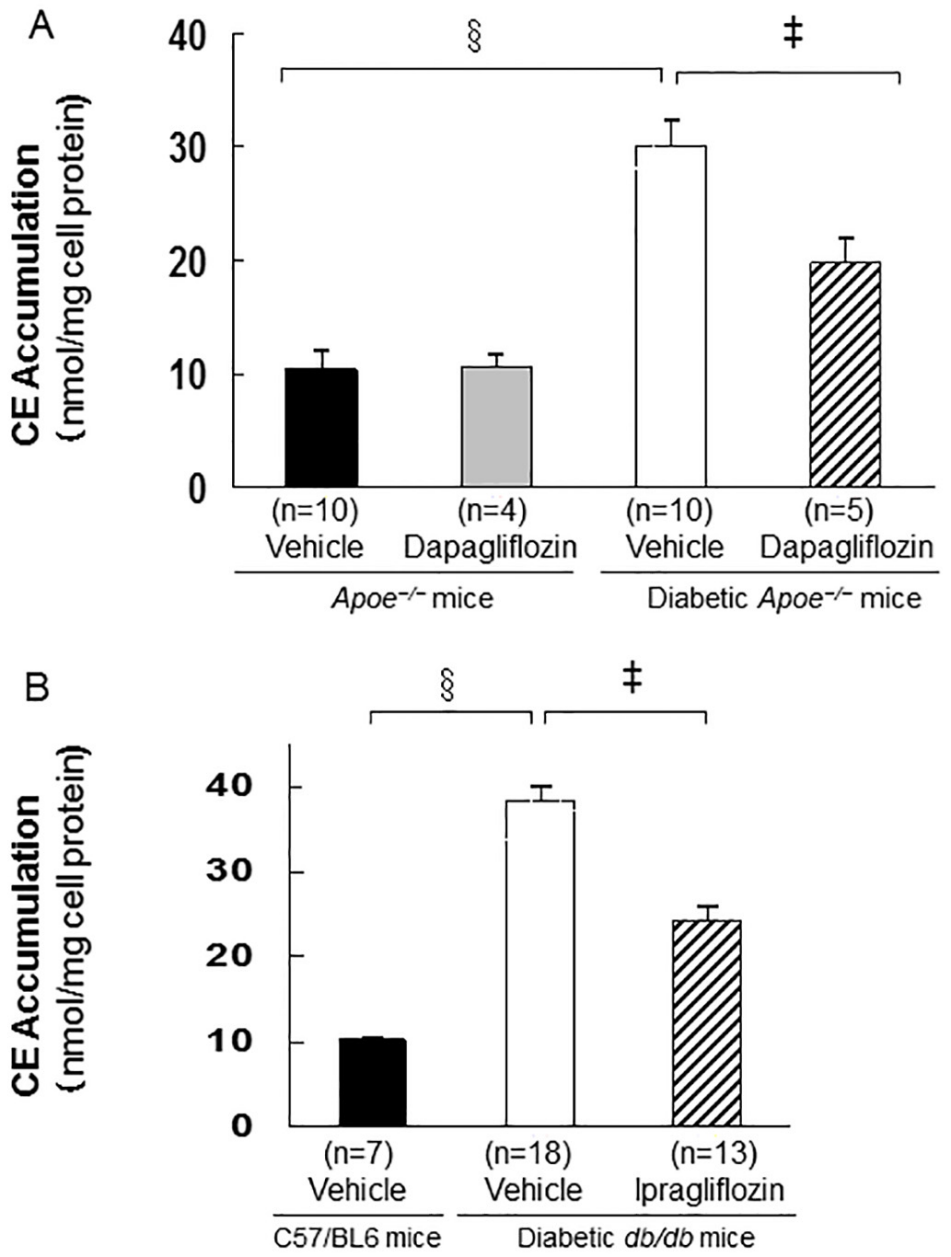
Foam cell formation in exudate peritoneal macrophages obtained from non-diabetic and diabetic *Apoe* ^*−/−*^ mice (A), and diabetic *db/db* mice (B). Four days after an intraperitoneal injection of thioglycolate, the exudated peritoneal cells were isolated from the treated non-diabetic and diabetic *Apoe* ^*−/−*^ mice at 21 weeks of age (Fig 3A), or from the diabetic *db/db* mice at 13 weeks of age (Fig 3B). Adherent macrophages were incubated for 18 hours with the RPMI-1640 medium containing 10μg/mL oxidized low-density lipoprotein (LDL) in the presence of 0.1 mmoL [^3^H]olate that was conjugated with bovine serum albumin. The cellular lipids were extracted and the radioactivity of the cholesterol [^3^H]olate was determined with thin-layer chromatography. Foam cell formation was expressed as cholesteryl ester (CE) accumulation. The values show mean ± SEM. One-way ANOVA followed by Tukey test: ^‡^ *p* < 0.001, ^§^ *p* < 0.0001.

The number of exudate peritoneal cells and morphological cell characteristics did not differ significantly among the groups. The ox-LDL-induced cholesterol ester accumulation, which indicated the degree of foam cell formation, was approximately 3–4 folds higher in macrophages obtained from both types of diabetic mice than in those obtained from non-diabetic mice (*p* < 0.0001; Fig 3A and 3B). This increased cholesterol ester (CE) accumulation in diabetic *Apoe* ^*−/−*^ mice was significantly suppressed with dapagliflozin treatment (34%, *p* < 0.001; Fig 3A). Similarly, ipragliflozin administration significantly reduced cholesterol ester accumulation in macrophages from diabetic *db/db* mice (31%, *p* < 0.001; Fig 3B).

### Correlation between atherosclerosis and glycemic control

**Fig 4:**
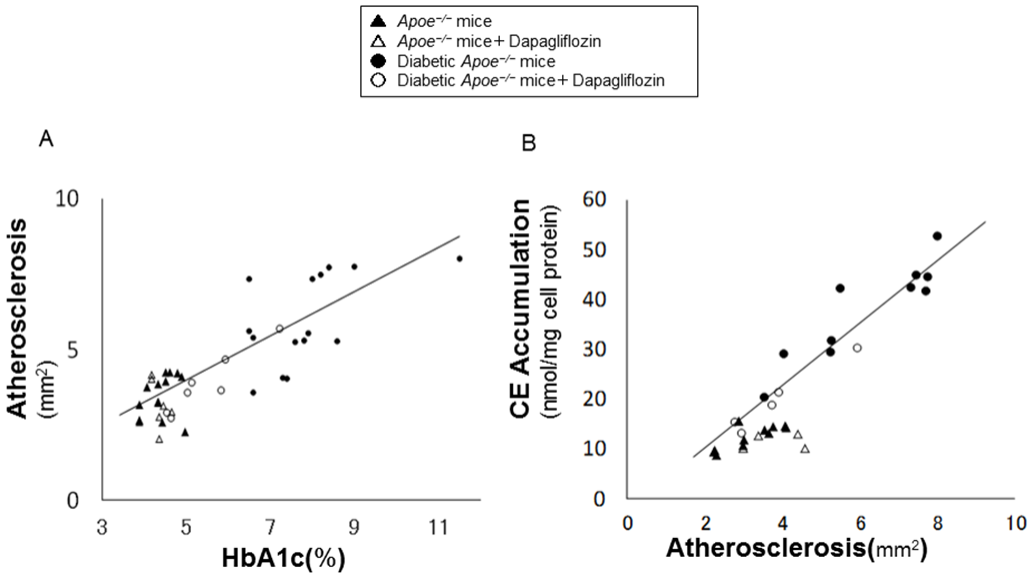
Correlation of atherosclerosis to glycemic control or foam cell formation in non-diabetic and diabetic *Apoe* ^*−/−*^ mice. Fig 4A shows the correlation between atherosclerosis and HbA1c in non-diabetic and diabetic *Apoe* ^*−/−*^ mice. Diabetic mice that received vehicle (n = 15); r = 0.56, *p* < 0.01. Diabetic mice that received dapagliflozin (n = 7); r = 0.94, *p* < 0.005. Combined diabetic mice (n = 22); r = 0.77, *p* < 0.0001. Fig 4B shows the correlation between foam cell formation and atherosclerosis in non-diabetic and diabetic *Apoe* ^*−/−*^ mice. Diabetic mice that received vehicle (n = 10); r = 0.91, *p* < 0.0005. Diabetic mice that received dapagliflozin (n = 5); r = 0.98, *p* < 0.005. Diabetic mice combined (n = 15); r = 0.95, *p* < 0.0001. *r* values indicate Pearson correlation coefficients. Pearson’s correlation test, *p* < 0.05.

**Fig 5:**
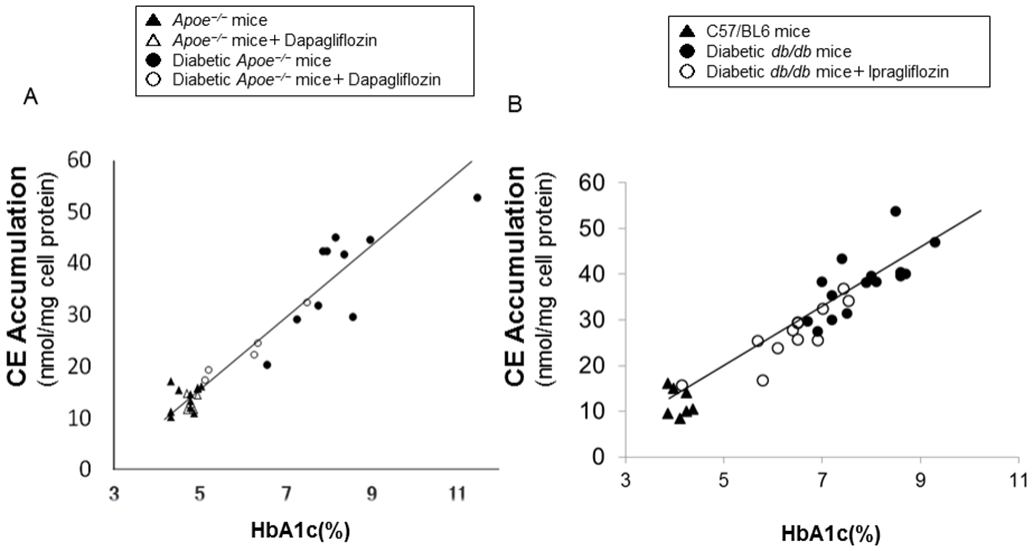
Correlation between foam cell formation and glycemic control in diabetic *Apoe* ^*−/−*^ mice (A) or diabetic *db/db* mice (B). Fig 5A shows the correlation between foam cell formation and HbA1c in non-diabetic and diabetic *Apoe* ^*−/−*^ mice. Diabetic mice that received vehicle (n = 10); r = 0.77, *p* < 0.01. Diabetic mice that received dapagliflozin (n = 5); r = 0.98, *p* < 0.005. Combined diabetic mice (n = 15); r = 0.91, *p* < 0.0001. Fig 5B shows the correlation between foam cell formation and HbA1c in diabetic *db/db* mice and wild-type (C57/BL6) mice. Diabetic mice that received vehicle (n = 18); r = 0.71, *p* < 0.001. Diabetic mice that received ipragliflozin (n = 13); r = 0.87, *p* < 0.0001. Combined diabetic mice (n = 31); r = 0.88, *p* < 0.0001. *r* values indicate Pearson correlation coefficients. Pearson’s correlation test, *p* < 0.05.

To examine the relationship between the development of atherosclerosis and glycemic control, we tested the correlation between atherosclerotic lesions and HbA1c levels. In diabetic *Apoe* ^*−/−*^ mice, atherosclerotic lesions highly correlated with HbA1c levels (r = 0.77, p < 0.0001, n = 22). However, no correlation was observed in the non-diabetic group (Fig 4A). In the analysis of the correlation between foam cell formation and atherosclerosis, diabetic *Apoe* ^*−/−*^ mice showed a strong correlation (r = 0.95, *p* < 0.0001, n = 15), while non-diabetic mice did not (Fig 4B). We also evaluated the correlation between the degrees of foam cell formation and glycemic control. The test demonstrated a significant correlation between foam cell formation and HbA1c levels only in diabetic *Apoe* ^*−/−*^ mice (r = 0.91, *p* < 0.0001, n = 15) and in diabetic *db/db* mice (r = 0.88, *p* < 0.0001, n = 31) but not in non-diabetic mice (Fig 5A and 5B). In addition, we examined the correlation of atherosclerosis or foam cell formation with various metabolic parameters other than glucose in diabetic *Apoe* ^*−/−*^ mice and diabetic *db/db* mice (Table 3). Body weight, systolic blood pressure, insulin, total cholesterol, or triglyceride did not correlate with the atherosclerotic lesion area or the degree of foam cell formation in either type of diabetic animals.

**Table 3:** Correlation of atherosclerosis or foam cell formation to metabolic parameters

	Diabetic *Apoe* ^*−/−*^ mice				Diabetic *db/db* mice	
	Atherosclerotic lesions		Foam cell formation		Foam cell formation	
	(n = 22)		(n = 15)		(n = 31)	
	r	p value	r	p value	r	p value
Body weight	−0.07	0.74	−0.09	0.74	0.28	0.14
SBP	0.26	0.21	0.34	0.19	0.06	0.79
Insulin	−0.3	0.3	−0.33	0.34	−0.15	0.44
Total-C	0.31	0.12	0.16	0.55	−0.27	0.18
Triglyceride	0.32	0.13	0.24	0.38	2.0	0.29

Total diabetic *Apoe* ^*−/−*^ mice that received vehicle or dapagliflozin, total diabetic *db/db* mice that received vehicle or ipragliflozin were measured.

SBP, systolic blood pressure; BW, body weight; Total-C, total cholesterol. *r* values indicate Pearson correlation coefficient. *p*< 0.05.

### Gene expressions in exudate peritoneal macrophages

**Fig 6:**
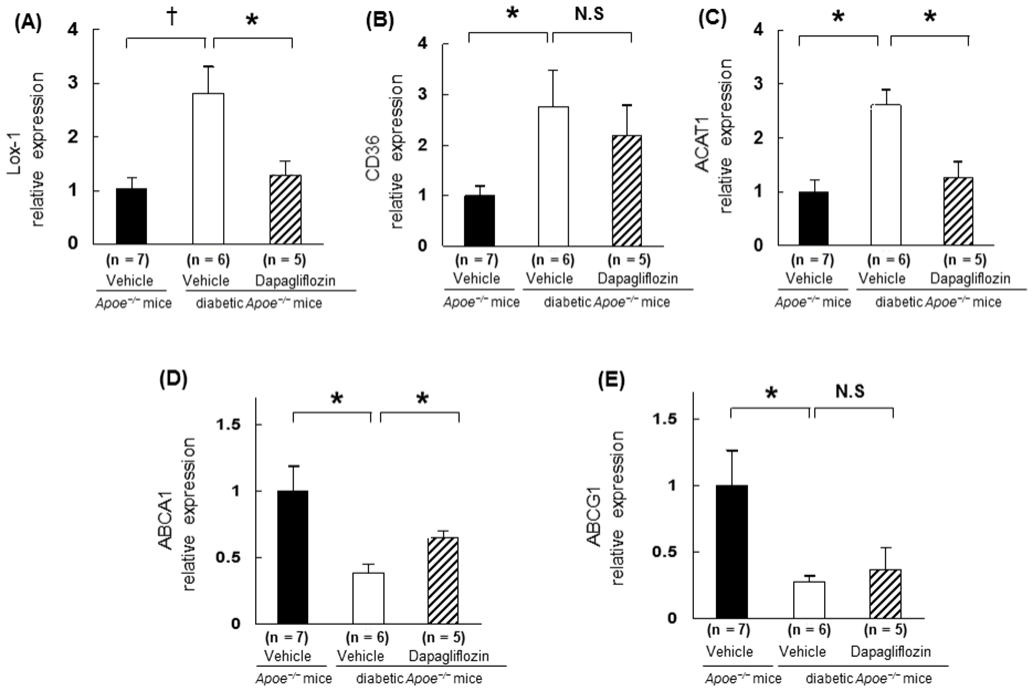
Changes in gene expression related to foam cell formation in the macrophages obtained from *Apoe* ^*−/−*^ mice. Gene expressions were measured in the peritoneal macrophages obtained from non-diabetic *Apoe* ^*−/−*^ mice (n = 7), diabetic *Apoe* ^*−/−*^ mice that received vehicle (n = 6), and those that received dapagliflozin (n = 5). Gene expressions of (A) lectin-like ox-LDL receptor-1 (Lox-1), (B) CD36, (C) acyl-coenzyme A:cholesterol acyltransferase 1 (ACAT1), (D) ATP-binding cassette transporter A1 (ABCA1), and (E) ATP-binding cassette sub-family G member1 (ABCG1) and the association with GAPDH were analyzed by real-time RT-PCR before the addition of ox-LDL. The values show mean ± SEM. One-way ANOVA followed by Tukey test for the comparison of *Apoe* ^*−/−*^ mice: *p < 0.05, ^†^ *p* < 0.01.

**Fig 7:**
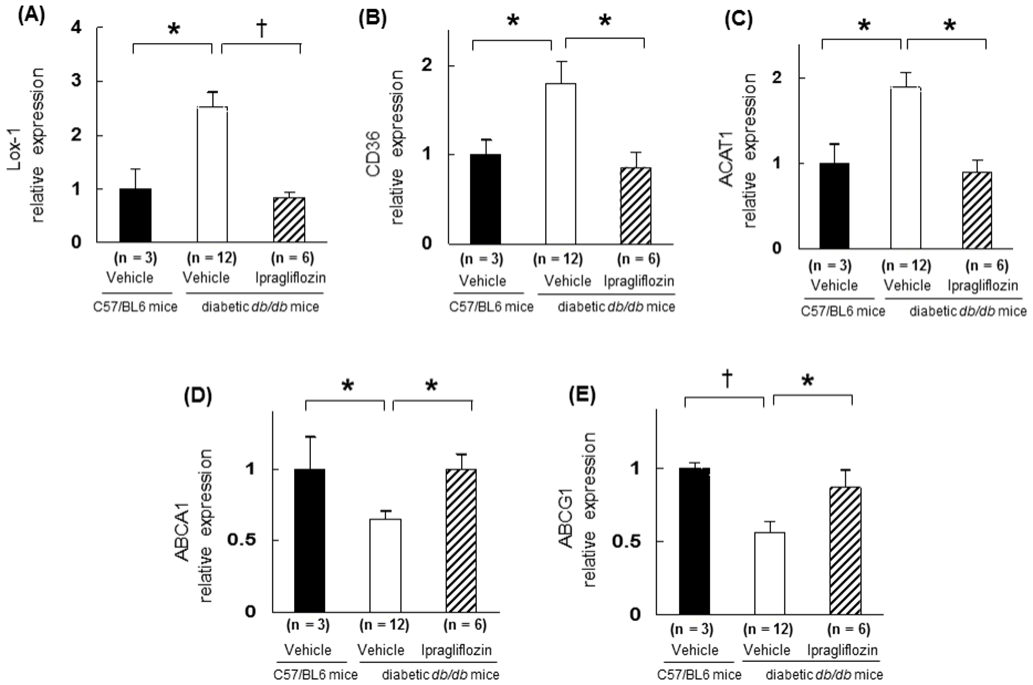
Changes in gene expression related to foam cell formation in the macrophages obtained from *db/db* mice and wild-type mice (C57/BL6). Gene expressions were measured in the peritoneal macrophages obtained from wild-type (C57/BL6) mice (n = 3), diabetic *db/db* mice that received vehicle (n = 12), and those that received ipragliflozin (n = 6). Gene expressions of (A) Lox-1, (B) CD36, (C) ACAT1, (D) ABCA1, and (E) ABCG1 in association with GAPDH were analyzed by real-time RT-PCR before the addition of ox-LDL. The values show mean ± SEM. One-way ANOVA followed by Tukey test: *p < 0.05, ^†^ *p* < 0.01.

To explore molecular changes related to macrophage foam cell formation in a diabetic state, we analyzed the expression of selected genes in peritoneal macrophages. The macrophages from both diabetic *Apoe* ^*−/−*^ mice and diabetic *db/db* mice showed that the expressions of Lox-1, CD36, and ACAT1 were elevated, in contrast the expressions of ABCA1 and ABCG1 were reduced compared with those in non-diabetic mice (*p* < 0.05; Figs 6 and 7). Dapagliflozin treatment reversed these changes except for CD36 and ABCG1 (*p* < 0.05; Fig 6). The gene expressions of Lox-1, CD36, and ACAT1 were upregulated, while those of ABCA1 and ABCG1 were downregulated in *db/db* diabetic mice. Ipragliflozin treatment normalized these abnormal gene expressions that had facilitated foam cell formation (*p* < 0.05; Fig 7).

## Discussion

This study was the first to demonstrate that SGLT2i prevented the development of atherosclerotic lesions in diabetic *Apoe* ^*−/−*^ mice. Moreover, the present study revealed a strong relationship between HbA1c levels and atherosclerotic lesions or macrophage foam cell formation. A strikingly close relationship between atherosclerotic lesions and foam cell formation may suggest a causative role for foam cell formation in atherogenesis. Because diabetic *db/db* mice do not accelerate visible atherosclerotic lesions, macrophage foam cell formation was used as a surrogate marker for atherosclerosis in this study. We also found that macrophage foam cell formation was substantially increased in diabetic *db/db* mice and that SGLT2i attenuated this in an HbA1c-dependent manner. To the best of our knowledge, the present study was the first to indicate a strong relationship between glycemia and macrophage foam cell formation *ex vivo*. The assay for foam cell formation usually requires 2–3 mice to obtain enough peritoneal macrophages for completion. We successfully measured foam cell formation in material obtained from one mouse, which enabled us to determine the individual associations of foam cell formation with HbA1c levels or atherosclerotic lesion area.

We used two types of diabetic mice whose insulin secretion and insulin resistance levels were completely in opposition. However, SGLT2i uniformly suppressed macrophage foam cell formation in both mice. Considering the findings in non-obese STZ-induced diabetic mice and the nature of this agent, it is reasonable to assume that lowering glucose levels can attenuate atherosclerosis independent of any insulin actions. SGLT2i significantly reduced body weight only in obese *db/db* mice but not in STZ-induced diabetic lean mice. Because body weight changes were not correlated with foam cell formation in diabetic *db/db* mice, weight loss itself may not be a major factor for the anti-atherosclerotic effects of SGLT2i. However, there is a possibility that improvement of insulin sensitivity could have indirectly contributed to suppression of atherosclerosis or macrophage form cell formation. Insulin resistance plays an important role in development and progression of atherosclerosis. Previous studies demonstrate that a SGLT2i improved insulin sensitivity in patients with type 2 diabetes [29]. We conducted oral glucose loading for assessment of glucose tolerability. However, the results did not indicate insulin sensitivity, and the lack of evaluation of insulin sensitivity is one of the limitations in this study. Further study using euglycemic-hyperinsulinemic clamp test with tracer is necessary to clarify change of insulin sensitivity by a SGLT2i and its role in atherosclerosis. Severe hypercholesterolemia may cause atherosclerosis in mice, such as in *Apoe* ^*−/−*^ mice. As in a previous study [24], plasma cholesterol levels were somewhat higher in diabetic *Apoe* ^*−/−*^ mice than in non-diabetic *Apoe* ^*−/−*^ mice and SGLT2i tended to lower these levels. However, there was no significant correlation between cholesterol levels and atherosclerotic lesion area or foam cell formation in either non-diabetic or diabetic *Apoe* ^*−/−*^ mice that did or did not receive SGLT2i. Therefore, it is unlikely that a mild reduction in cholesterol by SGLT2i can account for a substantial anti-atherosclerotic effect of SGLT2i. Similarly, plasma triglyceride, HDL cholesterol, and blood pressure were unaltered by SGLT2i in *Apoe* ^*−/−*^ mice and diabetic *db/db* mice; this indicated that changes in other risk factors for atherosclerosis outside of glycemia could not account for the favorable effect of SGLT2i on atherosclerosis. Thus, the pure glucose lowering effect of SGLT2i may lead to the suppression of atherogenesis in both type 1 and type 2 diabetic animals.

Macrophage CD36 scavenges ox-LDL, which leads to foam cell formation, and appears to be a key proatherogenic molecule [1, 30, 31]. The gene expression of CD36 in macrophages was substantially upregulated in both type 1 and type 2 diabetic mice. There have been supportive data from studies that demonstrated that CD36 expression or protein was highly expressed in diabetic mice [31, 32] and that high glucose induced CD36 expression by stimulating the translational process in cultured macrophages [31–33]. The upregulated gene expression of CD36 was attenuated by SGLT2i treatment in macrophages that were derived from diabetic *db/db* mice and corresponded to its glucose-lowering effect. However, an insignificant suppressive effect of SGLT2i on CD36 expression was observed in diabetic *Apoe* ^*−/−*^ mice, although there was a glucose-lowering effect that was similar to the one attained in diabetic *db/db* mice. On the other hand, another scavenger receptor, Lox-1 expression, was completely attenuated by SGLT2i in both diabetic mice models. Li *et al* reported that the gene expression of Lox-1 in macrophages was elevated by high glucose levels, which was directly linked to foam cell formation [17]. Moheimani *et al* reported that high glucose levels increased Lox-1 expression but the researchers failed to observe any upregulation of CD36 expression [34]. Lox-1 may be more glucose sensitive than CD36. Nevertheless, the majority of previous studies have supported that high glucose levels induced scavenger receptors in macrophages, which facilitated foam cell formation [17, 18, 31].

Macrophage foam cell formation characterized by cholesterol ester accumulation is modulated by ACAT1 [1], a rate-limiting enzyme for the esterification of free cholesterol from ox-LDL. Our data showed that ACAT1 gene expression was strongly enhanced in macrophages from type 1 and type 2 diabetic mice, and the amelioration of hyperglycemia by SGLT2i attenuated ACAT1 expression. However, because ACAT1 expression is regulated by the substrate (free cholesterol) availability inside cells [35], a change in ACAT1 might simply be a consequence of changes in scavenger receptors. Further studies will be required to clarify the direct relationship between glucose and ACAT1 independent of scavenger receptors.

The cholesterol efflux system plays a pivotal role in the suppression of foam cell formation in macrophages. ABCA1 and ABCG1 are the main molecules for cholesterol efflux from macrophage to apolipoprotein A-1 or HDL particles [1, 36–39]. There have been conflicting results in terms of ABCA1 and ABCG1 expression in macrophages derived from non-diabetic/diabetic mice or in high glucose treatment [19, 20, 40]. Mauldin *et al* reported that diabetic *db/db* macrophages had impaired cholesterol efflux to HDL but not to lipid-free apo A-1; this suggested that the increased levels of esterified cholesterol in the diabetic *db/db* macrophages were due to a selective loss of ABCG1-mediated efflux to HDL [41]. On the other hand, several studies have reported that ABCA1 expression was also downregulated by high glucose [19, 42, 43]. In the present study, a change in glycemia seems to more strongly influence ABCA1 expression than ABCG1 expression. Because we did not compare the actual cholesterol efflux to apolipoprotein A1 or HDL, it remains unknown whether upregulated ABCA1 by SGLT2i contributed to the correction of glucose-induced macrophage foam cell formation.

Polyol pathway flux, advanced glycation end-product (AGE) formation, protein kinase C (PKC) activity, and hexosamine pathway flux have all been shown to be involved in hyperglycemia-induced vascular damage [44]. In addition, chronic exposure to oxidative stress induces systemic inflammation [45, 46]. Given the fact that a number of studies support the crucial role of inflammation in the pathogenesis of atherosclerosis and the strong association between hyperglycemia and systemic inflammation, amelioration of hyperglycemia by SGLT2i might have also contributed to suppression of systemic inflammation via reduced ROS, subsequently resulting in protection against atherosclerosis. As a limitation of this study, changes in inflammation or ROS were not investigated. Our study should be extended to identify other possible mechanisms for glucose-stimulated macrophage foam cell formation and its related atherogenesis.

## Conclusions

Our study is the first to demonstrate that SGLT2i exerts anti-atherogenic effects by the normalization of blood glucose levels in both mouse models of type 1 and type 2 diabetes through suppressing macrophage foam cell formation; this is independent of insulin action. Our results suggest that foam cell formation regulated by Lox-1, ACAT1, and ABCA1 may be highly sensitive to glycemia *ex vivo*.

## Abbreviations

SGLT2: Sodium-glucose cotransporter 2
ABCA: ATP-binding cassette transporter A
ABCG: ATP-binding cassette sub-family G member
ACAT: Acyl-coenzyme A:cholesterol acyltransferase
CD36: Cluster of Differentiation 36
Lox: Lectin-like oxLDL receptor
GLP-1: Glucagon-like peptide-1
GIP: Glucose-dependent insulinotropic polypeptide
DPP4i: Dipeptidyl peptidase-4 inhibitor
*Apoe*^−/−^ mice: Apolipoprotein E-null mice
HbA1c: Hemoglobin A1c
LDL: Low-density lipoprotein
ox-LDL: Oxidized LDL
HDL: High-density lipoprotein
OGTT: Oral glucose tolerance test
ANOVA: Analysis of variance

## References

[1] Allahverdian S, Pannu PS, Francis GA. Contribution of monocyte-derived macrophages and smooth muscle cells to arterial foam cell formation. *Cardiovasc Res*. 2012; 95: 165–172. 10.1093/cvr/cvs094 22345306

[2] Lusis AJ. Atherosclerosis. *Nature*. 2000; 407: 233–241. 11001066 10.1038/35025203 PMC2826222

[3] Glass CK, Witztum JL. Atherosclerosis. the road ahead. *Cell*. 2001; 104: 503–516. 11239408 10.1016/s0092-8674(01)00238-0

[4] Nathan DM, Cleary PA, Backlund JY, Genuth SM, Lachin JM, Orchard TJ, et al Intensive diabetes treatment and cardiovascular disease in patients with type 1 diabetes. *N Engl J Med*. 2005; 353:2643–2653. 16371630 10.1056/NEJMoa052187 PMC2637991

[5] Turnbull FM, Abraira C, Anderson RJ, Byington RP, Chalmers JP, Duckworth WC, et al Intensive glucose control and macrovascular outcomes in type 2 diabetes. *Diabetologia*. 2009; 52: 2288–2298. 10.1007/s00125-009-1470-0 19655124

[6] Chait A, Bornfeldt KE. Diabetes and atherosclerosis: is there a role for hyperglycemia?. *Journal of Lipid Research*. 2009; 50 (supplement): S335–339. 19029122 10.1194/jlr.R800059-JLR200 PMC2674740

[7] Ford ES, Zhao G, Li C. Pre-diabetes and the risk for cardiovascular disease: a systematic review of the evidence. *J Am Coll Cardiol*. 2010; 55: 1310–1317. 10.1016/j.jacc.2009.10.060 20338491

[8] Gami AS, Witt BJ, Howard DE, Erwin PJ, Gami LA, Somers VK, et al Metabolic syndrome and risk of incident cardiovascular events and death: a systematic review and meta-analysis of lomgitudinal studies. *J Am Coll Cardiol*. 2007; 49: 403–414. 17258085 10.1016/j.jacc.2006.09.032

[9] Mottillo S, Filion KB, Genest J, Joseph L, Pilote L, Rinfret S, et al The metabolic syndrome and cardiovascular risk a systematic review and meta-analysis. *J Am Coll Cardiol*. 2010; 56: 1113–1132. 10.1016/j.jacc.2010.05.034 20863953

[10] Chen GP, Zhang XQ, Wu T, Li L, Han J, Du CQ. Alteration of mevalonate pathway in proliferated vascular smooth muscle from diabetic mice: possible role in high-glucose-induced atherogenic process. *J Diabetes Res*. 2015: 379287 10.1155/2015/379287 25918730 PMC4396976

[11] Mudaliar H, Pollock C, Ma J, Wu H, Chadban S, Panchapakesan U. The role of TLR2 and 4-mediated inflammatory pathways in endothelial cells exposed to high glucose. *PLoS One*. 2014; 9: e108844 10.1371/journal.pone.0108844 25303153 PMC4193767

[12] Hamuro M, Polan J, Natarajan M, Mohan S. High glucose induced nuclear factor kappa B mediated inhibition of endothelial cell migration. *Atherosclerosis*. 2002; 162: 277–287. 11996947 10.1016/s0021-9150(01)00719-5

[13] Ferrannini E, Solini A. SGLT2 inhibition in diabetes mellitus: rationale and clinical prospets. *Nat Rev Endocrinol*. 2012; 8: 495–502. 10.1038/nrendo.2011.243 22310849

[14] DeFronzo RA, Hompesch M, Kasichayanula S, Liu X, Hong Y, Pfister M, et al Characterization of renal glucose reabsorption in response to dapagliflozin in healthy subjects and subjects with type 2 diabetes. *Diabetes Care*. 2013; 36: 3169–3176. 10.2337/dc13-0387 23735727 PMC3781504

[15] Nauck MA. Update on develioments with SGLT2 inhibitors in the management of type 2 diabetes. *Drug Des Devel Ther*. 2014; 8: 1335–1380. 10.2147/DDDT.S50773 25246775 PMC4166348

[16] Fujita Y, Inagaki N. Renal sodium glucose cotransporter 2 inhibitors as a novel therapeutic approach to treatment of type 2 diabetes: Clinical data and mechanism of action. *J Diabetes Investig*. 2014; 5: 265–275. 10.1111/jdi.12214 24843771 PMC4020327

[17] Li L, Sawamura T, Renier G. Glucose enhances human macrophage LOX-1 expression: role for LOX-1 in glucose-induced macrophage foam cell formation. *Circulation Res*. 2004; 94:892–901. 15001526 10.1161/01.RES.0000124920.09738.26

[18] Fukuhara T, Sakai M, Sakamoto Y, Takeya M, Horiuchi S. Expression of class A scavenger receptor is enhanced by high glucose in vitro and under diabetic conditions in vivo: one mechanism for an increased rate of atherosclerosis in diabetes. *J Bio Chem*. 2005; 280, 3355–3364. 15556945 10.1074/jbc.M408715200

[19] Chang YC, Sheu WH, Chien YS, Tseng PC, Lee WJ, Chiang AN. Hyperglycemia accelerates ATP-binding cassette transporter A1 degradation via an ERK-dependent pathway in macrophages. *Journal of Cellular Biochemistry*. 2013; 114:1364–1373. 10.1002/jcb.24478 23239052

[20] Mauerer R, Ebert S, Langmann T. High glucose, unsaturated and saturated fatty acids differentially regulate expression of ATP-binding cassette transporters ABCA1 and ABCG1 in human macrophages. *Exp Mol Med*. 2009; 41:126–132. 19287193 10.3858/emm.2009.41.2.015 PMC2679329

[21] Takeda Y, Fujita Y, Honjo J, Yanagimachi T, Sakagami H, Takiyama Y, et al Reduction of both beta cell death and alpha cell proliferation by dipeptidyl peptidase-4 inhibition in a streptozotocin-induced model of diabetes in mice. *Diabetologia*. 2012; 55: 404–412. 10.1007/s00125-011-2365-4 22072158

[22] Graham ML, Janecek JL, Kittredge JA, Hering BJ, Schuurman HJ. The streptozotocin-induced diabetic nude mouse model: differences between animals from different sources. *Comp Med*. 2011; 61:356–360. 22330251 PMC3155402

[23] Terasaki M, Nagashima M, Watanabe T, Nohtomi K, Mori Y, Miyazaki A, et al Effects of PKF275-055, a dipeptidyl peptidase-4 inhibitor, on the development of atherosclerotic lesions in apolipoprotein E-null mice. *Metabolism*. 2012; 61:974–977. 10.1016/j.metabol.2011.11.011 22225957

[24] Terasaki M, Nagashima M, Nohtomi K, Kohashi K, Tomoyasu M, Sinmura K, et al Preventive effect of dipeptidyl peptidase-4 inhibitor on atherosclerosis is mainly attributable to incretin’s actions in nondiabetic and diabetic apolipoprotein E-null mice. *PLoS One*. 2013; 8: e70933 10.1371/journal.pone.0070933 23967137 PMC3742603

[25] Nogi Y, Nagashima M, Terasaki M, Nohtomi K, Watanabe T, Hirano T. Glucose-dependent insulinotropic polypeptide prevents the progression of macrophage-driven atherosclerosis in diabetic apolipoprotein E-null mice. *PLoS One*. 2012; 7: e35683 10.1371/journal.pone.0035683 22536426 PMC3335006

[26] Nagashima M, Watanabe T, Terasaki M, Tomoyasu M, Nohtomi K, Kim-Kaneyama J, et al Native incretins prevent the development of atherosclerotic lesions in apolipoprotein E knockout mice. Diabetologia. 2011; 54:2649–2659. 10.1007/s00125-011-2241-2 21786155 PMC3168747

[27] Nagashima M, Watanabe T, Shiraishi Y, Morita R, Terasaki M, Arita S, et al Chronic infusion of salusin-alpha and -beta exerts opposite effects on atherosclerotic lesion development in apolipoprotein E-deficient mice. *Atherosclerosis*. 2010; 212: 70–77. 10.1016/j.atherosclerosis.2010.04.027 20684826

[28] Arora S, Ojha SK, Vohora D. Characterisation of Streptozotocin Induced Diabetes Mellitus in Swiss Albino Mice. *Global Journal of Pharmacology*. 2009; 3: 81–84.

[29] Merovci A, Mari A, Solis C, Xiong J, Daniele G, Chavez-Velazquez A, et al Dapagliflozin improves muscle insulin sensitivity but enhances endogenous glucose production. *J Clin Invest*. 2014; 124: 509–514. 10.1172/JCI70704 24463448 PMC3904617

[30] Lin CS, Lin FY, Ho LJ, Tsai CS, Cheng SM, Wu WL, et al PKCδsignaling regulates SR-A and CD36 expression and foam cell formation. *Cardiovascular Res*. 2012; 95: 346–355. 10.1093/cvr/cvs189 22687273

[31] Griffin E, Re A, Hamel N, Fu C, Bush H, McCaffrey T, et al A link between diabetes and atherosclerosis: Glucose regulates expression of CD36 at the level of translation. *Nature Medicine*. 2001; 7, 840–846. 11433350 10.1038/89969

[32] Kaplan M, Kerry R, Aviram M, Hayek T. High glucose concentration increases macrophage cholesterol biosynthesis in diabetes through activation of the sterol regulatory element binding protein 1 (SREBP1): inhibitory effect of insulin. J *Cardiovasc Pharmacol*. 2008; 52: 324–332. 10.1097/FJC.0b013e3181879d98 18791464

[33] Hayek T, Hussein K, Aviram M, Coleman R, Keidar S, Pavoltzky E, et al Macrophage foam-cell formation in streptozotocin-induced diabetic mice: stimulatory effect of glucose. *Atherosclerosis*. 2005; 183:25–33. 16216589 10.1016/j.atherosclerosis.2005.02.018

[34] Moheimani F, Tan JT, Brown BE, Heather AK, van Reyk DM, Davies MJ. Effect of exposure of human monocyte-derived macrophages to high, versus normal, glucose on subsequent lipid accumulation from glycated and acetylated low-density lipoproteins. *Exp Diabetes Res* 2011; 2011: 851280 10.1155/2011/851280 21904540 PMC3166758

[35] Sekiya M, Osuga J, Igarashi M, Okazaki H, Ishibashi S. The role of neutral Cholesterol Ester Hydrolysis in Macrophage Foam Cells. *Journal of Atherosclerosis and Trombosis*. 2011; 18: 359–364. 10.5551/jat.7013 21467808

[36] Kennedy MA, Barrera GC, Nakamura K, Baldan A, Tarr P, Fishbein MC, et al ABCG1 has a critical role in mediating cholesterol efflux to HDL and preventing cellular lipid accumulation. *Cell Metab*. 2005; 1:121–131. 16054053 10.1016/j.cmet.2005.01.002

[37] Oram JF, Vaughan AM. ATP-binding cassette cholesterol transporters and cardiovascular disease. *Circ Res*. 2006; 99: 1031–1043. 17095732 10.1161/01.RES.0000250171.54048.5c

[38] Wang X, Collins HL, Ranalletta M, Fuki IV, Billheimer JT, Rothblat GH, et al Macrophage ABCA1 and ABCG1, but not SR-BI, promote macrophage reverse cholesterol transport in vivo. *J Clin Invest*. 2007; 117: 2216–2224. 17657311 10.1172/JCI32057 PMC1924499

[39] Yvan-Chravet L, Wang N, Tall AR. Role of HDL, ABCA1 and ABCG1 transporters in cholesterol efflux and immune response. *Atheroscler Thromb Vasc Biol*. 2010; 30: 139–143. 10.1161/ATVBAHA.108.179283 PMC2812788 19797709

[40] Machado-Lima A, Iborra RT, Pinto RS, Castilho G, Sartori CH, Oliveira ER, et al In type 2 diabetes mellitus glycated albumin alters macrophage gene expression impairing ABCA1-mediated cholesterol efflux. *J of Cellular physiology*. 2015; 230: 1250–1257. 10.1002/jcp.24860 25413254

[41] Mauldin JP, Srinivasan S, Mulya A, Gebre A, Parks JS, Daugherty A, et al Reduction in ABCG1 in Type 2 diabetic mice increases macrophage foam cell formation. *J Bio Chem*. 2006; 281: 21216–21224. 16723355 10.1074/jbc.M510952200

[42] Passarelli M, Tang C, McDonald TO, O’Brien KD, Gerrity RG, Heinecke JW, et al Advanced glycation end product precursors impair ABCA1-dependent cholesterol removal from cells. *Diabetes*. 2005; 54: 2198–2205. 15983222 10.2337/diabetes.54.7.2198

[43] Patel DC, Albrecht C, Pavitt D, Paul V, Pourreyron C, Newman SP, et al Type 2 diabetes is associated with reduced ATP-binding cassette transporter A1 gene expression, protein and function. *PLoS One*. 2011; 6: e22142 10.1371/journal.pone.0022142 21829447 PMC3144880

[44] Brownlee M. Biochemistry and molecular cell biology of diabetic complications. *Nature*. 2001; 414: 813–820. 11742414 10.1038/414813a

[45] Piarulli F, Sartore G, Lapolla A. Glyco-oxidation and cardiovascular complications in type 2 diabetes: a clinical update. *Acta Diabetol*. 2013; 50: 101–110. 10.1007/s00592-012-0412-3 22763581 PMC3634985

[46] Evans JL, Goldfine ID, Maddux BA, Grodsky GM. Oxidative stress and stress-activated signaling pathways: a unifying hypothesis of type 2 diabetes. *Endocr Rev*. 2002; 23: 599–622. 12372842 10.1210/er.2001-0039

